# Development and characterisation of a large diameter decellularised vascular allograft

**DOI:** 10.1007/s10561-017-9673-y

**Published:** 2017-11-29

**Authors:** A. Aldridge, A. Desai, H. Owston, L. M. Jennings, J. Fisher, P. Rooney, J. N. Kearney, E. Ingham, S. P. Wilshaw

**Affiliations:** 1 Institute of Medical and Biological Engineering, School of Biomedical Sciences, The University of Leeds, Leeds, LS2 9JT UK; 2 Institute of Medical and Biological Engineering, School of Mechanical Engineering, The University of Leeds, Leeds, LS2 9JT UK; 3 NHS Blood and Transplant Tissue and Eye Services, 14 Estuary Banks, Estuary Commerce Park, Speke, Liverpool, L24 8RB UK

**Keywords:** Decellularisation, Acellular, Scaffold, Aorta

## Abstract

The aims of this study were to develop a biological large diameter vascular graft by decellularisation of native human aorta to remove the immunogenic cells whilst retaining the essential biomechanical, and biochemical properties for the ultimate benefit of patients with infected synthetic grafts. Donor aortas (n = 6) were subjected to an adaptation of a propriety decellularisation process to remove the cells and acellularity assessed by histological analysis and extraction and quantification of total DNA. The biocompatibility of the acellular aortas was determined using standard contact cytotoxicity tests. Collagen and denatured collagen content of aortas was determined and immunohistochemistry was used to determine the presence of specific extracellular matrix proteins. Donor aortas (n = 6) were divided into two, with one half subject to decellularisation and the other half retained as native tissue. The native and decellularised aorta sections were then subject to uniaxial tensile testing to failure [axial and circumferential directions] and suture retention testing. The data was compared using a paired t-test. Histological evaluation showed an absence of cells in the treated aortas and retention of histoarchitecture including elastin content. The decellularised aortas had less than 15 ng mg^−1^ total DNA per dry weight (mean 94% reduction) and were biocompatible as determined by in vitro contact cytotoxicity tests. There were no gross changes in the histoarchitecture [elastin and collagen matrix] of the acellular aortas compared to native controls. The decellularisation process also reduced calcium deposits within the tissue. The uniaxial tensile and suture retention testing revealed no significant differences in the material properties (*p* > 0.05) of decellularised aorta. The decellularisation procedure resulted in minimal changes to the biological and biomechanical properties of the donor aortas. Acellular donor aorta has excellent potential for use as a large diameter vascular graft.

## Introduction

Large diameter vascular grafts have been used successfully to treat a number of conditions, including aortic aneurysms and aortic dissections. Abdominal aortic aneurysms affect 4–7% of men and 1–2% of women over the age of 65 years (Robinson et al. 2013). Aortic dissection is the most serious complication of thoracic aortic disease and has been shown to affect from 6 to 16.3 in 100,000 cases per year (Howard et al. 2013; Olsson et al. 2006).

Currently, the preferred treatment option for aortic replacement is to insert a readily available synthetic graft, most commonly a woven Dacron^®^ or expanded polytetrafluoroethylene; ePTFE graft. Aortic replacement with Dacron^®^ prostheses is performed widely with acceptable outcomes and has a relatively low rate of post-implantation and graft related complications. Infection rates of synthetic grafts in open surgery can, however be as high as 6% (Cernohorsky et al. 2011; Heyer et al. 2009; Vicaretti et al. 1998) and although the incidence of infection in endovascular aortic repair (EVAR) surgery is scarce (< 1%) the recommended treatment for infected synthetic grafts remains the same; an open surgical procedure involving wide debridement of the infected vessel and the surrounding tissue, followed by extra-anatomic revascularisation with long term antibiotics (Swain et al. 2004; Ting et al. 2005; Davila et al. 2015).

In the setting of an infected synthetic aortic graft, a cryopreserved human allograft provides an alternative method of reconstruction for patients. Historically, allografts were prone to disintegration, aneurysmal degeneration and rupture post-operatively and this limited their use in early cases of arterial reconstruction following infection (Szilagyi et al. 1970). The use of cryopreserved heart valves in aortic arch and cardiac valve reconstruction (O’Brien et al. 2001; Sabik et al. 2002) however rekindled the exploration of other vascular locations that may be suitable for cryopreserved vascular allografts.

The use of vascular allografts may however lead to allosensitization (Benedetto et al. 2001; Wilshaw et al. 2012). Aortic allograft conduits (and heart valves) have been shown to undergo degenerative changes, including calcification, following implantation. Calcification and degeneration is believed to be caused by the immune response towards donor cells (Baskett et al. 2003; Dignan et al. 2003; Muratov et al. 2010; Webb et al. 1992), and can result in the failure of the graft. Moreover, vascular allografts may exhibit inherent calcification. This is due to the dystrophic calcification that occurs in vascular tissue due to aging, atherosclerosis and aortic stenosis. Calcification of the aortic wall occurs as a part of the aging process in the majority of people. Vascular allografts will therefore likely exhibit calcification to some degree, and this will be detrimental to the function and performance of the graft since calcium deposits may act as foci for additional calcification.

Since cryopreserved allografts contain donor cells with associated antigens which initiate an adverse host response and act as foci for calcification it is logical to propose that removal of the cellular components of allografts would reduce the immune response and calcification. The aim of decellularisation of allograft tissue is to remove immunogenic cells, creating biocompatible grafts without significantly affecting the structural components of the extracellular matrix and biomechanical properties. This approach has been successfully used to produce decellularised heart valve allografts which have been used clinically (Bechtel et al. 2005; Brown et al. 2010; Burch et al. 2010; Cebotari et al. 2011; Ruzmetov et al. 2012; Tavakkol et al. 2005; Zehr et al. 2005). There are, however, no studies that have investigated the inherent calcification present in donor cardiovascular tissues before and after decellularisation.

We hypothesize that decellularisation, together with removal of calcium, from aortic allografts will improve life-span and durability when compared to current cryopreserved aortic allografts. Here we describe the application of a proprietary decellularisation process (Wilshaw et al. 2012), based on the use of low concentration sodium dodecyl sulphate (SDS) to aortic allografts. In order to reduce the inherent calcium content of the donor aortas, an initial decalcification treatment using ethylenediaminetetraacetic acid (EDTA) was incorporated into the process. Decellularisation of aortic allograft is an attractive approach since retention of the histo-architecture of the native aorta may allow constructive remodelling by host cells to generate a living, functional graft.

## Materials and methods

### Tissue procurement

Human aortas were obtained from UK NHS Blood and Transplant Tissue and Eye Services. The study was carried out under full UK NHS ethical approval (08/H0405/65). The aortas were obtained within 48 h of death. The tissues were stored and transported at − 80 °C. The aortas were of thoracic origin, between 8 and 20 cm in length and varied in internal diameter from 1.5 to 3.0 cm. Excess fat and connective tissue were removed and the adventitial surface of the aortas was scraped using a scalpel blade prior to washing in phosphate-buffered saline (PBS; Oxoid). The tissue used in this study was provided from male donors aged between 45 and 79.

### Decellularisation

The decellularisation process was modified from Wilshaw et al. (2012) for human donor common femoral arteries. The decellularisation process was carried out using aseptic technique throughout. The aortas were thawed at 40 °C for 60 min and only aortas with no grossly visible atherosclerotic plaques or calcification were used. Aortas were subsequently washed twice in a 10% (w/v) ethylenediaminetetraacetic acid (EDTA; VWR International) solution with 10 KIU mL^−1^ Aprotinin (Mayfair House) in water for 24 h at 40–45 °C. The aortas were then sequentially washed in hypotonic Tris buffer (10 mM Tris; Sigma-Aldrich, 0.1%; v/v; EDTA, and 10 KIU mL^−1^ Aprotinin, pH 8.0) at 40–45 °C for 24 h; 0.1% (v/v) sodium dodecyl sulphate (SDS; Sigma-Aldrich) in Tris hypotonic buffer at 40–45 °C for 24 h; PBS twice for 15 min at 42 °C before a 72 h wash at 40–45 °C in PBS. The tissues were treated with nuclease (50 mM tris, 1 mM MgCl_2_·6H_2_0, 10 U mL^−1^ Benzonase (Merck)) for 3 h twice before subsequent washes in PBS twice for 15 min at 42 °C followed by a 24 h wash at 40–45 °C. Aortas were decontaminated in Cambridge antibiotic solution (Source Bioscience) for 3 h at 37 °C followed by washes in PBS twice for 15 min at 42 °C followed by a 24 h wash at 40–45 °C. The aortas were washed using hypertonic buffer solution (1.5 M sodium chloride (ThermoFisher Scientific Ltd.) and 50 mM Tris, pH 7.5) for 24 h at 40–45 °C. Final washes in PBS included two washes for 15 min at 42 °C followed by a 24 h wash at 40–45 °C and a final 72 h at 40–45 °C. This resulted in a 14 day decellularisation process.

### Histological evaluation

Native and decellularised tissue samples from each of six different human aortas were fixed in zinc fixative solution (0.5% w/v zinc chloride (VWR Chemicals), 0.5% w/v zinc acetate (VWR Chemicals), in 0.1 M Tris buffer containing 0.05% w/v calcium acetate (Sigma) at pH 7.4) for 48 h, dehydrated, and embedded in paraffin wax using a Leica Tissue Processor (TP1020). Serial sections (6–8 µm) were stained with haematoxylin and eosin (H&E), picrosirius red/Millers elastin (to visualize collagen and elastin) and alcian blue (glycosaminoglycans, GAGs) using standard histological methods. Cell nuclei were visualised using DAPI dye (Sigma Aldrich); briefly, sections of tissue were rehydrated in a graded alcohol series before they were incubated in the dark in 0.1 µg mL^−1^ DAPI dye in dye buffer (10 mM Tris, 1 mM EDTA, 1 mM sodium chloride in water) for 10 min. The sections were viewed using an upright Zeiss Imager M2 microscope under normal Kohler illumination or under fluorescent illumination using a DAPI filter (λ_ex_ = 340–380 nm/λ_em_ = 435–485 nm) and images were captured digitally using Zen Blue Pro (Zeiss).

### Calcium detection and quantification

Sections of native and decellularised zinc fixed tissue were subject to von Kossa staining. Briefly, sections of tissue were rehydrated in a graded alcohol series before staining in a 1% w/v silver nitrate (AnalarR) solution in water for 1 h on a Kenro Light Box (2 × 15 W bulbs). Following this the samples were washed in a 158 mM sodium thiosulphate pentahydrate (VWR) solution in water for 5 min before rinsing in water and counterstaining with eosin for 30 s. Calcium quantification was performed using a commercial kit (Abcam; AB102505). Samples of tissue for calcium quantification were lyophilised to a constant weight using a Modulo D freeze dryer (Thermo) before being hydrolysed in 6 M hydrochloric acid for 6 h at 120 °C, at 15 lb sqin^−1^ and subsequent neutralization with 6 M sodium hydroxide. The samples were then assayed according to the manufacturer’s recommendations. Calcium content was quantified by interpolation of the absorption of the sample data from a known set of calcium standards (Abcam; AB102505).

### DNA extraction and quantification

Total DNA was extracted from human native and decellularised tissues (n = 6) using a DNeasy blood and tissue kit from Qiagen. Finely macerated tissue (~ 25 to 50 mg wet weight native tissue or ~ 100 to 150 mg wet weight decellularised tissue) was lyophilised to a constant weight, and the freeze dried tissue digested using proteinase K at 56 °C for 16 h. RNAse was used to remove any RNA present within the samples. The samples were washed by centrifugation to remove contaminating matter. Finally, the DNA was eluted into Eppendorf tubes and quantified using a NanoDrop spectrophotometer at 260 nm (Thermo Fisher Scientific).

### In-vitro biocompatibility assays

#### Cell culture

 A549 adenocarcinomic human alveolar basal epithelial cells (ECACC) were cultured in Dulbecco’s modified Eagle’s medium (DMEM) containing 10% (v/v) FBS, 100 U mL^−1^ penicillin, 100 mg mL^−1^ streptomycin, and 100 mM l-glutamine (Lonza) at 37 °C in 5% CO_2_ (v/v) in air. BHK baby hamster kidney cells (ECACC) were cultured in Glasgow’s modified Eagle’s medium (GMEM) containing 5% (v/v) FBS, 10% (v/v) tryptose phosphate broth (Oxoid) at 37 °C in 100 U mL^−1^ penicillin, 100 mg mL^−1^ streptomycin, and 100 mM l-glutamine at 37 °C in 5% CO_2_ (v/v) in air.

#### Contact cytotoxicity assay

Samples of decellularised human aorta (5 mm^2^; n = 6) were attached to the centre of the wells of a six-well culture plate using steri-strips (Medisave). Cyanoacrylate glue was used as positive control, and steri strips alone were used as the negative control. A549 or BHK cells were seeded into each well at a density that allowed 80% confluence after cell seeding. Plates were incubated at 37 °C in 5% (v/v) CO_2_ in air for 48 h. The cells were fixed with 10% (v/v) neutral buffered formalin (Genta Medica) for 10 min prior to staining with Giemsa solution (VWR International) for 5 min. The culture wells were carefully rinsed with distilled water until the water ran clear and air dried before examining cell morphology and distribution under normal Kohler illumination.

### Immunohistochemistry

Native and decellularised zinc fixed and paraffin embedded tissue sections (from n = 6 human aortas) were stained with monoclonal antibodies against collagen I (Millipore; IgG isotype, polyclonal, 1:500 dilution), collagen III (Millipore; IgG1 isotype, clone IE7-D7, 1:100 dilution), collagen IV (Dako; IgG1 isotype, clone PHM-12, 1:200 dilution), von Willebrand factor (Dako; IgG isotype, polyclonal, 1:400 dilution), fibronectin (Vector; IgG1 isotype, clone 568, 1:150 dilution) and laminin (Sigma; IgG1 isotype, clone LAM-89, 1:1000 dilution). Immunolabeling was carried out using an Ultravision detection system (Thermo Scientific). Antigen retrieval was employed using proteinase K (Dako; room temperature for 20 min—collagen III, von Willebrand factor, fibronectin and laminin), proteinase K followed by trypsin digestion (0.05% w/v trypsin 0.52 mM EDTA.4Na in HBSS with phenol red (Sigma) for 30 s) (collagen IV) and Vector antigen unmasking solution citric based (collagen I). Hydrogen peroxide (Sigma) was used to block endogenous peroxidase activity. Isotype control antibodies (IgG and IgG1; Dako; used at the same concentrations as the test antibody) and omission of the primary antibody served as negative controls. Sections were viewed using Kohler and all images were captured digitally using Zen Blue Pro (Zeiss).

### Biomechanical testing

Paired samples of native and decellularised aorta were generated by halving native aorta before the decellularisation process, with half undergoing decellularisation and the remaining native aorta utilised in the biomechanical assays detailed below (uniaxial tensile testing and suture pull-out testing).

### Uniaxial tensile testing

Paired native (n = 6) and decellularised (n = 6) human aorta tissue samples were subjected to low strain rate uniaxial loading to failure. Native and decellularised human aorta were cut open along their length and rectangular aortic tissue strips with width 5 mm were cut in both axial and circumferential orientations from each native and decellularised human aorta. The tissue was cut using a custom-made cutter comprising of two Stanley knife blades held a fixed 5 mm apart. Prior to testing, the thickness of the samples was measured at six points along the gauge length using a digital thickness gauge (PK-0510, Mitutoyo UK Ltd) with a resolution of 0.01 mm, and their average thickness was calculated. Subsequently, the samples were mounted onto a custom made holder. The holder was supported by a removable bracket that allowed alignment of the two holder grips, pre-defining the gauge length of the specimen at 10 mm, and ensuring that no load was imposed onto the specimen until the test began. Once the test sample was clamped onto the holder, the holder with supporting bracket was secured to an Instron 3365 (Instron^®^ Corporation) uniaxial tensile tester with 50 N load cell and the supporting bracket was removed. The samples were tested at 10 mm min^−1^ under hydrated conditions in PBS at 37 °C. The load and extension data was recorded during tests. The load-extension data were converted into stress–strain data from which the ultimate tensile stress (UTS), elastin phase slope and collagen phase slope were determined.

Suture pull-out testing: Samples of native (n = 6) and decellularised (n = 6) aorta were dissected into rectangular specimens of 20 mm length and 15 mm width. A 4-0 non-absorbable monofilament suture (Premilene^®^) was placed into the free end of the tissue, 4 mm from the end to form a loop. Prior to testing, the thickness of the samples was measured near the suture hole using a thickness gauge (as above). Subsequently, the samples were mounted in an Instron 3365 (Instron^®^ Corporation) uniaxial tensile tester with a 50 N load cell. The tissue was clamped using one set of the grips, the other set of grips were used to clamp the looped suture. A uniaxial load was applied to the suture until it was pulled out of the specimen, with strain rate 10 mm min^−1^. The maximum force (N) required to pull-out the suture was recorded.

### Determination of collagen and denatured collagen content

Hydroxyproline (as an indirect measure of collagen) assays were performed on samples of lyophilised native and decellularised human aortas (n = 6).

#### Hydroxyproline assay

Samples were hydrolysed in 6 M hydrochloric acid for 6 h at 120 °C, at 15 lb sqin^−1^ and subsequently neutralized with 6 M sodium hydroxide. The hydroxyproline assay was performed as described by Edwards and O’Brien (1980). The hydroxyproline content was determined by interpolation of sample data from a trans-4-hydroxy-l-proline standard curve. The total collagen content was calculated by using a hydroxyproline to collagen ratio of 1:7.14 (Harding and Wesley 1968).

#### Denatured collagen assay

The assay was performed as described by Bank et al. (1997). Samples were treated with 5 mg mL^−1^ α-chymotrypsin (Sigma) in 0.1 M tris buffer at pH 7.8 containing 2.5 mM calcium chloride for 24 h at 30 °C. The samples were centrifuged at 600×*g* for 10 min. The supernatant was hydrolysed, and then neutralized and the hydroxyproline content was determined as described above.

### Statistical analysis

All numerical data were analysed using Graphpad Prism 6 and all data were presented as mean ± 95% confidence intervals (CI). The Student’s t-test and paired samples t-test was used for comparison of two group means as appropriate.

## Results

### Development of the decellularisation process for human donor aortas

**Fig. 1:**
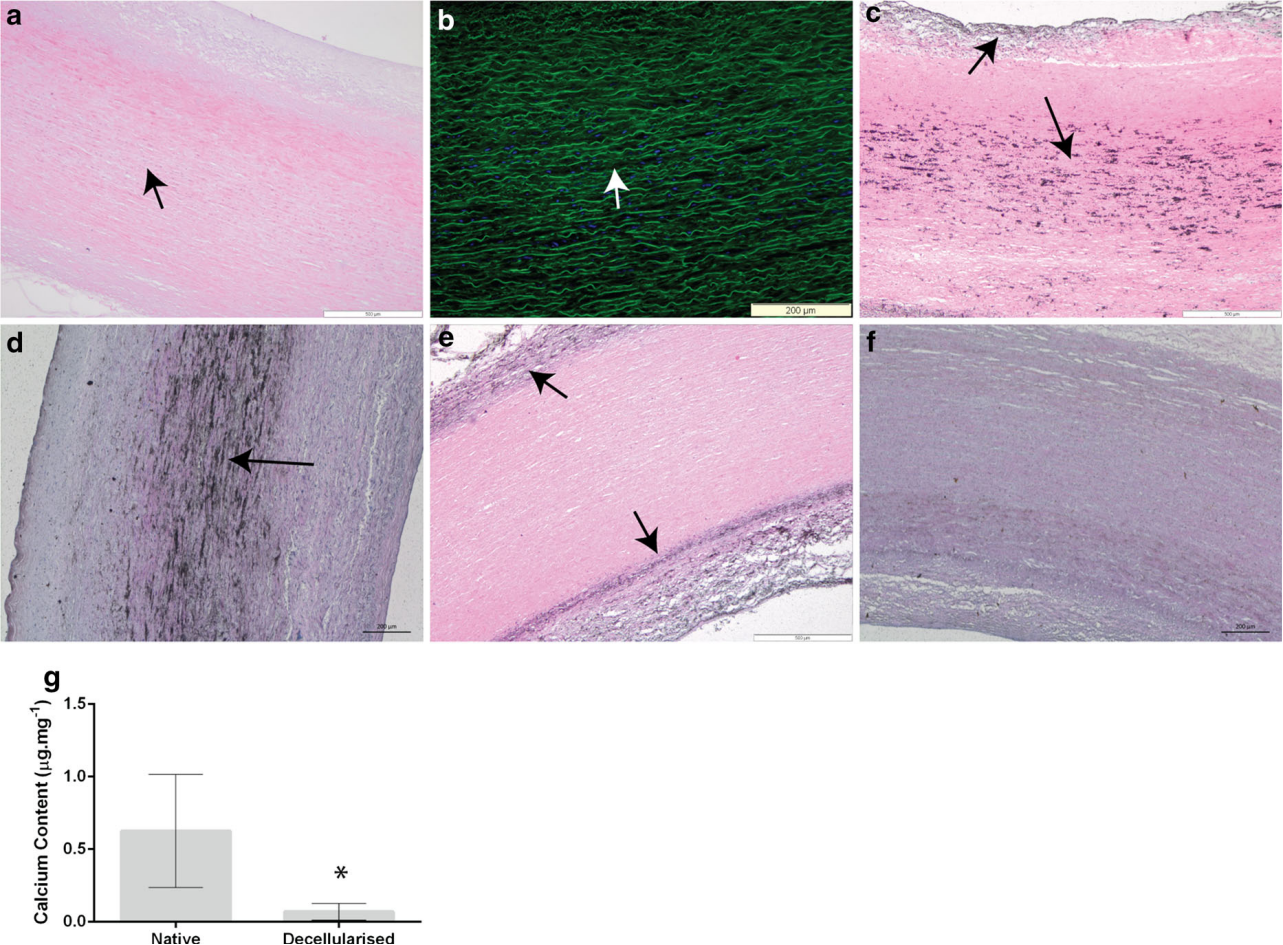
Sections of human aorta stained using haematoxylin and eosin (**a**) and DAPI (**b**). Sections stained using DAPI were viewed using fluorescence microscopy, the cell nuclei appeared blue (λ_ex_ 365 nm/λ_em_ 445 nm) and the ECM green (λ_ex_ 470 nm/λ_em_ 525 nm), black/white arrows denotes remaining cell nuclei following decellularisation. **c**, **d** Sections of native human aorta stained using Von Kossa’s stain. **e** Sections of decellularised human aorta, processed using initial decellularisation protocol, stained using Von Kossa’s stain, black arrows highlight regions of calcification. **f** Von Kossa staining of decellularised human aorta decellularised using the revised protocol. Images were viewed and acquired using normal Kohler illumination and a × 20 objective, scale bars represent 100 µm. **g** quantification of the calcium content of fresh and acellular human aorta, data represents mean (n = 6) ± 95% confidence intervals. *Significantly different as determined using two tailed, unpaired Students t-test, *p* < 0.005

When the original (Wilshaw et al. 2012) decellularisation process was applied to the human aortas, cellular remnants were frequently observed in H&E and DAPI stained sections of the tunica media (Fig. 1a, b). Since sections of the native human aortas showed the presence of calcium deposits when stained with von Kossa (Fig. 1c, d), it was hypothesized that incomplete decellularisation was due to calcification present in the donor aortas. The original decellularisation protocol failed to remove this calcium, and calcium deposits initially observed in the tunica media appeared to be washed into the intima and remaining adventitia during the decellularisation process (Fig. 1e). A decalcification step was therefore introduced to the start of the decellularisation process. von Kossa staining of aorta tissue sections from samples that had been decalcified and decellularised demonstrated greatly reduced calcium staining (Fig. 1f). Quantitation of the calcium content of human aortas before and after decalcification and decellularisation showed that native human aorta contained 0.63 ± 0.39 (95% CI) µg mg^−1^ calcium, which was reduced to 0.07 ± 0.06 µg mg^−1^ following the decalcification and decellularisation process (Fig. 1g).

### Histological evaluation of native and decellularised aortas

**Fig. 2:**
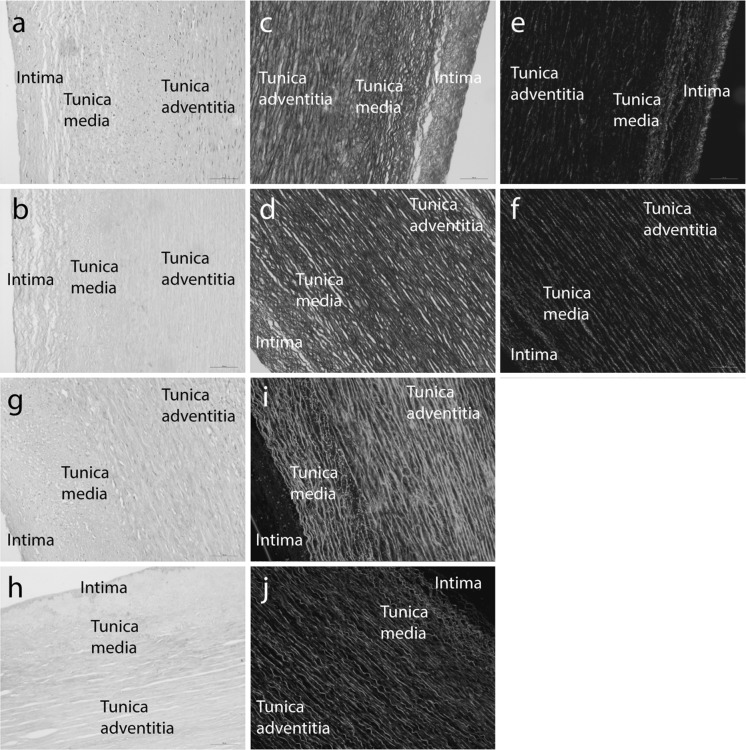
Sections of (**a**, **c**, **e**, **g**, **i**) native and (**b**, **d**, **f**, **h**, **j**) acellular human aorta stained using (**a**, **b**) Haematoxylin and eosin (**c**, **d**) Millers elastin (**e**, **f**) Sirius red (**g**, **h**) Alcian blue and (**i**, **j**) DAPI. Sections stained using DAPI were viewed using fluorescence microscopy, the cell nuclei appeared blue (λ_ex_ 365 nm/λ_em_ 445 nm) and the ECM green (λ_ex_ 470 nm/λ_em_ 525 nm). Images were acquired using a × 20 objective, scale bars represent 100 µm

The decalcification and decellularisation process was applied to a further six human donor aortas. There was no evidence of gross changes to the histioarchitecture of the aorta tissues following the process (Fig. 2a, b) and observations of H&E stained sections showed no evidence of cell nuclei or whole cells in the decellularised tissues. Sections of the native aorta stained with H&E showed the trilaminar structure; comprised of the intima, tunica media and the tunica adventitia (Fig. 2a, b). The majority of the adventitia had been removed by blunt dissection prior to decellularisation (Fig. 2b). Although these layers were visible in the sections stained using the range of histological staining methods, they were most obvious when the sections were stained with Sirius red and Miller’s elastin (Fig. 2c–f). Miller’s elastin and Sirius red predominantly stain collagen and elastin fibrils that are circumferentially orientated in native vessels (Fig. 2c, e) and these fibres and their orientation were retained following decellularisation (Fig. 2d, f). Alcian blue stained sections revealed the GAG content of both native and decellularised tissues, with no apparent loss of GAG staining following the decellularisation process (Fig. 2g, h). GAG staining was most intense around the endothelium. DAPI stained sections of decellularised aorta tissue sections demonstrated a lack of visible cell nuclei when compared to native control tissue sections (Fig. 2i, j).

### DNA content of native and decellularised aortas

The DNA was extracted from native and decellularised human aortas (n = 6) using a commercially available kit and quantified spectrophotometrically (260 nm). The DNA content of native aorta was 88 ± 43.4 (95% CI) ng mg^−1^ and acellular was 5 ± 1.5 ng mg^−1^ by dry weight. This represented a 94% reduction in DNA content between native and decellularised aorta.

### In-vitro biocompatibility of decellularised aortas

**Fig. 3:**
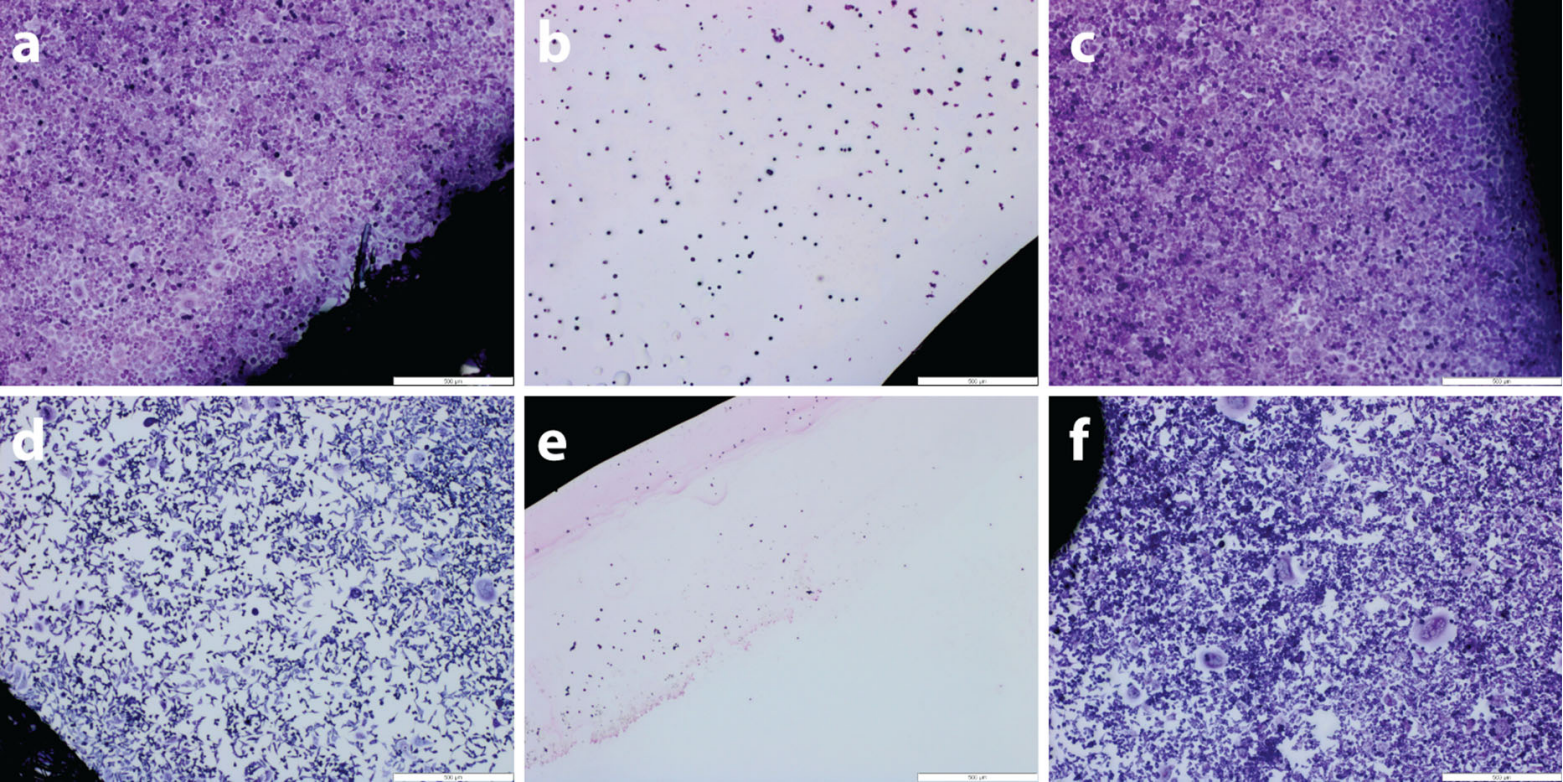
Contact cytotoxicity assessment of acellular human aorta, samples were cultured with human A549 lung epithelial cells. **a** negative control (steri-strip only), **b** positive control (cyanoacrylate) and **c** acellular human aorta. Additionally baby hamster kidney cells were used, **d** negative control (steri-strip only), **e** positive control (cyanoacrylate) and **f** acellular human aorta. The cell sheets were stained using Giemsa’s stain and all images acquired using a × 2 objective, the scale bars represent 500 µm

Microscopic analysis of the contact cytotoxicity assay plates demonstrated that A549 and BHK cells grew up to and in contact with samples of decellularised human aorta (Fig. 3). No zones of cell lysis or changes in cell morphology were apparent (Fig. 3). Steri-strip (negative control) was biocompatible and cyanoacrylate contact adhesive (positive control) caused cell lysis (Fig. 3).

### Immunohistochemical analysis of native and decellularised aorta

**Fig. 4:**
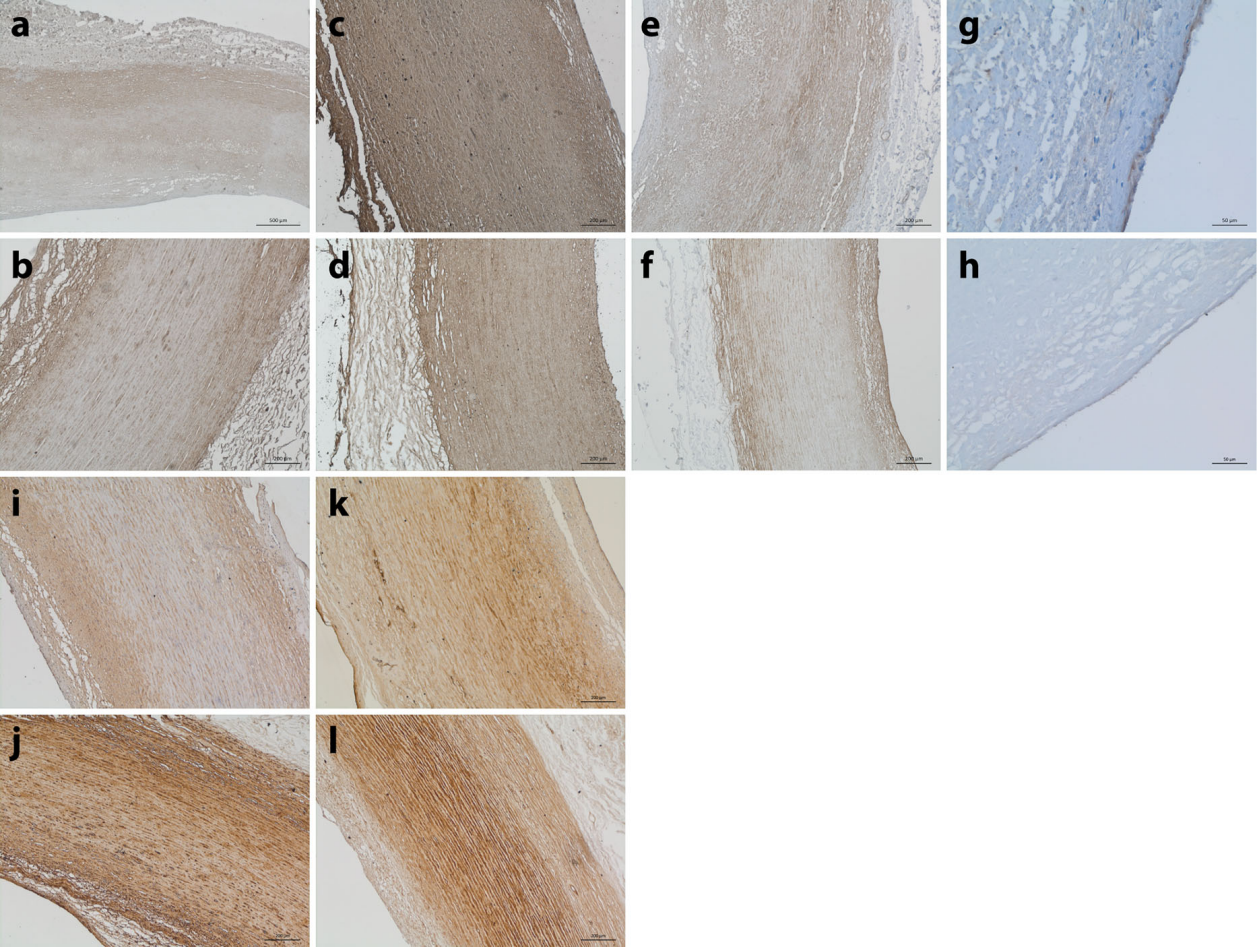
Sections of (**a**, **c**, **e**, **g**, **i**, **k**) native and (**b**, **d**, **f**, **h**, **j**, **l**) acellular human aorta labelled using monoclonal antibodies against (**a**, **b**) Collagen type I (**c**, **d**) Collagen type III (**e**, **f**) Collagen type IV (**g**, **h**) Von Willebrand factor, (**i**, **j**) Fibronectin and (**k**, **l**) Laminin. Images were acquired using Kohler illumination and a × 10 objective, scale bars represent 200 µm

Immunohistochemical staining of sections of decellularised human aorta demonstrated no loss of collagen type I and III when compared to sections of native tissue (Fig. 4a–d). Collagen type IV labelling was observed throughout the intima and tunica media, in both native and decellularised aortas, but was not present in the adventitia (Fig. 4e, f). Von Willebrand factor labelling was located in the endothelium and positive labelling was observed in both native and decellularised aorta (Fig. 4g, h). Fibronectin was labelled throughout sections of the tissues with no visible reduction in decellularised aorta compared to native aorta (Fig. 4i, j). Laminin was present throughout sections of the native and decellularised tissues, although there was a slight reduction of laminin labelling towards the luminal aspect of the tunica intima in decellularised tissue sections compared to native tissue sections (Fig. 4k, l).

### Biomechanical properties of native and decellularised aortas

The uniaxial tensile testing indicated no significant changes in the material properties of decellularised aorta tissue when compared to paired samples of native aorta. No significant differences were observed in collagen and elastin phase slopes and the ultimate tensile stress in either the circumferential or axial directions (paired samples t-test, *p* > 0.05; Table 1). All failures occurred at or near to the centre of the gauge length.

**Table 1:** Biomechanical parameters of native and decellularised human aorta

Sample (orientation)	Collagen phase slope (MPa)	Elastin phase slope (MPa)	Ultimate tensile stress (MPa)	Suture pull-out force (N)
Native (axial)	2.34 ± 0.95	0.31 ± 0.20	1.08 ± 0.35	6.09 ± 1.11
Decellularised (axial)	2.27 ± 1.52	0.50 ± 0.60	0.91 ± 0.47	6.98 ± 2.81
Native (circumferential)	3.14 ± 1.58	0.24 ± 0.17	1.43 ± 1.02	
Decellularised (circumferential)	3.86 ± 1.75	0.28 ± 0.17	1.33 ± 0.44	

Low strain rate loading to failure of decellularised and native aorta tissue in the circumferential and axial directions [data represents mean (n = 6) ± 95% confidence intervals, paired samples t-test, *p* > 0.05]. A suture was pulled out of samples of native and decellularised aortas to determine the maximum force (N) required to pull out the sutures [data represents mean (n = 6) ± 95% confidence intervals, paired samples t-test, *p* > 0.05]

During the suture pull-out testing, a suture was pulled out of the samples (native and decellularised) and the samples ruptured in the circumferential direction perpendicular to the direction of the testing. No significant differences were observed between the maximum suture pull-out force (N) required to pull out the sutures between native and decellularised aorta (paired samples t-test, *p* > 0.05; Table 1).

### Collagen and denatured collagen content of native and decellularised aortas

**Fig. 5:**
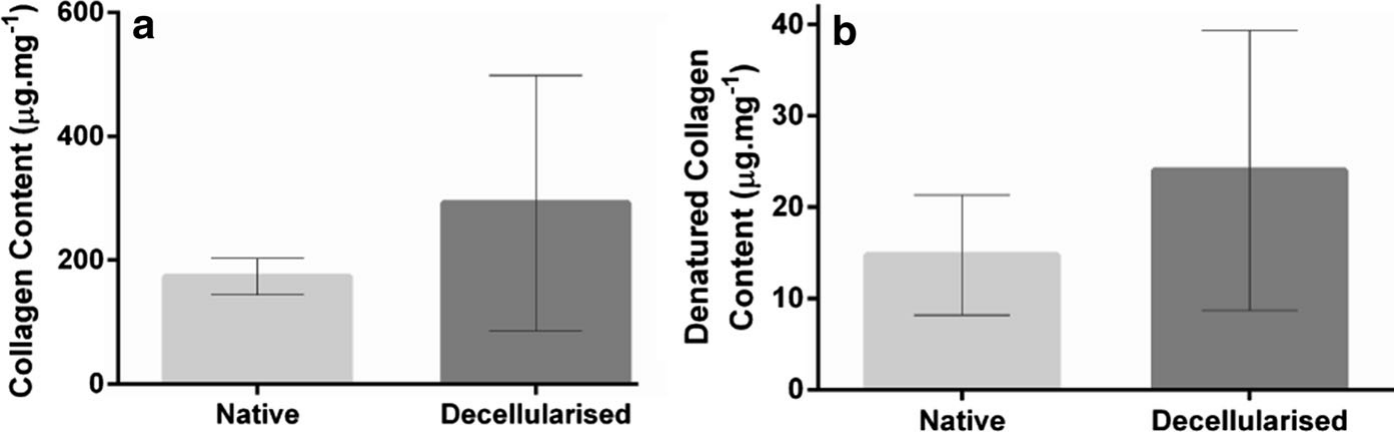
Collagen content of native and acellular human aorta. **a** Collagen content and **b** denatured collagen content. Data represents mean (n = 6) ± 95% confidence intervals. No significant differences between the properties of acellular arteries when compared to native controls as determined using Students t-test

The collagen content of native and decellularised aortas was 173.6 ± 29.4 (95% CI) and 292.6 ± 206.4 mg mL^−1^ respectively. The values were not significantly different (Student’s t-test, *p* = 0.18, Fig. 5a). There was no significant difference in the levels of denatured collagen of native and decellularised aortas (Student’s t-test, *p* = 0.26). The denatured collagen content of native and decellularised aorta was 14.8 ± 6.6 (95% CI) and 24.0 ± 15.33 mg mL^−1^ respectively (Fig. 5b).

## Discussion

There is a clinical need for a biological graft for the replacement of infected synthetic grafts used in the treatment of aortic dissection and aortic aneurism.

Studies have shown that decellularisation significantly reduces both the cellular and humoral immune response to allograft tissue (Dahl et al. 2011; Hilbert et al. 2004; Hopkins et al. 2009; Meyer et al. 2005). Clinically, the early and midterm results obtained with decellularised aortic valve allografts have shown good structural integrity, a low rate of calcification and no structural or morphological alteration (da Costa et al. 2010). Decellularised aortic valves present a promising alternative for aortic valve replacement in selected patients. For aortic grafts, cryopreserved aortas have been used in the clinic with reports of good long-term survival and patency (Gomez-Caro et al. 2008; Noel et al. 2002), but the presence of remaining donor cells/material in cryopreserved allografts may however lead to allosensitization (Benedetto et al. 2001; Wilshaw et al. 2012).

The aims of this study were to test the hypothesis that it was possible to decellularise human donor aorta tissues without adversely affecting the biomechanical or biological properties of the tissue. The decellularised aortas would then have the potential to be deployed in surgeries to replace infected synthetic aortic grafts, or indeed in primary aortic repair procedures without risk of an adverse host immune response.

The initial decellularisation process for human donor aortas was based on a protocol developed for allogeneic femoral arteries (Wilshaw et al. 2012). This process utilises hypotonic buffer in combination with low concentration SDS and nuclease enzymes to remove native cells whilst preserving the extracellular matrix. The use of detergents and osmotic shock is a common approach utilised to decellularise soft tissues (Crapo et al. 2011; Hogg et al. 2013; Luo et al. 2014; Wilshaw et al. 2012). Antibiotics were used to reduce the initial bioburden of the aortas as part of the decellularisation process. The initial decellularisation process, however resulted in acellular tissues with a significant calcium content. It was apparent that the donor aortas invariably exhibited calcification to some extent. This was most likely due to the dystrophic calcification that occurs in vascular tissue as a common complication in aging, atherosclerosis and aortic stenosis. When human aortic tissue ages the calcium content increases (Elliott and McGrath 1994; Tohno et al. 1998). The tissue used in this study was provided from donors aged between 45 and 79 and therefore exhibited significant calcification upon receipt.

The incorporation of a decalcification step using 10% (w/v) EDTA prior to decellularisation of the donor aortas enabled the production of tissues devoid of cells with no overt changes to the histo-architecture and circa 90% reduction in the calcium content. The residual calcium levels (70 mg mg^−1^ or 70 ppm) were in the region of those reported for decellularised 6-month old porcine aorta (91 ppm; Paniagua Gutierrez et al. 2015). Interestingly, alcian blue staining of tissue sections demonstrated no visible loss of GAGs. This was in contrast to other published studies that have noted a loss of GAG staining in various tissues decellularised using SDS (Luo et al. 2014; Stapleton et al. 2008; Xu et al. 2014).

The DNA content of the decellularised biological scaffolds is widely considered to be important in determining whether a decellularisation procedure is successful (Lotze et al. 2007; Nagata et al. 2010). Previous studies have shown that there is residual DNA present in existing commercially available biological scaffolds (Gilbert et al. 2009). Gilbert and colleagues used Picogreen™ assay to quantify *double stranded* DNA remaining following decellularisation. However the present study quantified total DNA using absorbance at 260 nm. The Picogreen assay is a more sensitive assay and can detect lower levels of DNA, but it is limited to detecting double stranded DNA. Utilising spectrophotometry to measure the absorbance of DNA in solution, both single stranded (and therefore partially digested DNA) as well as double stranded DNA was detected, allowing for true quantification of DNA content remaining in decellularised aorta. Following decellularisation there was an average of 5 ng mg^−1^ (dry weight) of DNA remaining, representing extremely low levels.

Collagen IV and laminin staining was present throughout the decellularised aorta, although laminin staining was reduced in the luminal regions, whereas staining with von Willebrand factor was localised to the luminal surface (basement membrane). The presence of von Willebrand staining in the basement membrane in the decellularised vessel demonstrated retention of at least one basement membrane protein Von Willebrand factor has been shown to be present in the extracellular matrix filaments and co-distributes with other components such as fibronectin and collagen IV (Handin and Wagner 1989; Wagner 1990). Laminin plays a role in the development of blood vessels. It has been suggested that reduction in laminin causes reduced innervation in implanted blood vessels in in vivo studies (Gavazzi et al. 1995; Hallmann et al. 2005), however additional in vivo implantation studies would be required to see if this would be the case with the decellularised aorta.

The decellularised aorta was biocompatible to cells in in vitro tests with no significant loss of collagen content or increase in denatured collagen. There was a trend to increased levels of collagen and denatured collagen in decellularised aortas compared to native aortas (not significant) which was most likely due to the change in tissue mass caused by the loss of cellular material. Importantly, there was no change in the biomechanical properties of the human aortas following decellularisation, demonstrating that the processing of the tissue preserved the structural integrity of the extracellular matrix.

Decalcification and decellularisation of human aorta therefore has the potential to retain the advantages of the currently available cryopreserved human arterial grafts while postponing or eliminating one of the quintessential markers for deterioration: calcification. Failure due to calcification is particularly troublesome when allografts are used in younger patients, and as such, an absence of calcium in the decellularised arterial grafts has the potential to provide an improved life-span and durability when compared to the currently used cryopreserved allografts. Before clinical use, however, it would be desirable to evaluate the acellular aorta in a functional animal model to determine patency.

## Conclusions

The data presented indicates that decalcified decellularised human aorta has the potential to be used as a large diameter vascular graft. The process developed was shown to be successful in removing both calcium and cells from the aortic tissue, with greater than 94% removal of total DNA. The decellularised aortas were cytocompatible and the structure of the aortas were maintained with no change to the biomechanical properties.
